# Computer Vision Syndrome Among Medical Students at the University of Khartoum, Sudan: Prevalence and Associated Factors

**DOI:** 10.7759/cureus.38762

**Published:** 2023-05-09

**Authors:** Hassan A Gadain Hassan

**Affiliations:** 1 Community Medicine, Faculty of Medicine, University of Khartoum, Khartoum, SDN

**Keywords:** virtual learning, electronic devices, medical students, ocular symptoms, digital eye strain, computer vision syndrome

## Abstract

Background and aims

Regular continuous uninterrupted use of electronic devices, such as smartphones, tablets, and computers, can result in a spectrum of vision-related symptoms known as computer vision syndrome. Students today can readily access information and books on their smartphones and computers, which reduces their reliance on printed texts. Numerous muscle-related and ocular complaints could arise from this. The primary objective of this study was to estimate the prevalence of computer vision syndrome symptoms among medical students at the University of Khartoum and to identify its contributing factors. The secondary objective was to evaluate practices and knowledge related to computer vision syndrome prevention.

Methods

This study is a facility-based cross-sectional observation aimed at describing medical students' characteristics at the University of Khartoum. The sampling strategy employed was stratified random sampling, and a structured online questionnaire was used to collect the data. A total of 149 students completed the self-administered questionnaire. The questionnaire included questions on sociodemographic data, validated symptoms of computer vision syndrome, and factors associated with the syndrome's development. Data were analyzed using SPSS Statistics (Armonk, NY: IBM Corp.), simple descriptive statistics were calculated, and odds ratios and Pearson’s chi-square test were employed to examine and quantify the association between variables.

Results

Of the 149 participants, 58.4% were female, while males made up 41.6% of the study sample. The prevalence of computer vision syndrome has been found to be 94%, and 72.4% of the students reported experiencing at least three symptoms of computer vision syndrome. Neck and shoulder pain was the most commonly reported symptom (78.5%), followed by headache (70.5%), while the least reported symptom was eye redness (36.2%). The majority of students (81.2%) used electronic devices for five or more hours a day, and the most common posture while using electronic devices was lying down, as reported by 54.4% of the students. A total of 68% of the medical students in this study reported keeping a distance that was shorter than the suggested 40 centimeters from the screen, and only 18.1% were aware of the 20-20-20 rule (every 20 minutes look at an object 20 feet away for 20 seconds). The seating position has been found to be significantly associated with the number of symptoms experienced (p=0.012); sitting with a bent back was 4.6 times more likely to cause more than three symptoms when compared to sitting upright with a straight back (OR=4.643; 95% CI: 1.63-13.21; p=0.004).

Conclusion

There was a very high prevalence of computer vision syndrome among medical students at the University of Khartoum. Most students had poor awareness and bad practices regarding the safe use of electronic devices. Awareness campaigns to encourage good practices and enable the safe use of computers and other digital devices are strongly recommended.

## Introduction

Electronic devices such as computers, tablets, iPads, and smartphones have become an essential part of university education. Because of Google, Wikipedia, and medical-related apps, smartphone use in education is fast growing. Students can readily locate resources and books on their computers and smartphones, reducing their reliance on paper-based reading materials. Computer vision syndrome (CVS) has the potential to become a major public health concern [1].

Computer vision syndrome is a collection of vision- and muscle-related symptoms brought on by prolonged continuous use of digital displays, including those found in computers, televisions, tablets, and smartphones, according to the American Optometric Association (AOA) [2]. Headache, eye strain, double vision, blurred vision, and dry eyes are some of the symptoms of CVS. It is well-established that prolonged use of computers is linked to musculoskeletal complaints, such as backaches, neck stiffness, and finger tingling and numbness [3].

Three following possible processes could be blamed for the symptoms of computer vision syndrome: (i) extra-ocular mechanisms, (ii) accommodative mechanisms, and (iii) ocular-surface mechanisms [4]. Extraocular mechanisms cause musculoskeletal symptoms like shoulder pain, backache, headache, and stiffness in the neck. These symptoms are closely linked to incorrect computer screen placement and improper postural habits, which result in muscle sprains [4]. Blurred vision, double vision, myopia, hypermetropia, and sluggish focus change are all brought on by accommodative mechanisms. After their shift ended, 20% of those who used computers displayed temporary nearsightedness, according to one study. Many persons may have minor accommodative or binocular issues that do not create symptoms when performing routine, less stressful visual tasks, but these problems are exacerbated by prolonged digital screen use [4]. Ocular surface mechanisms result in symptoms like dry eyes, gritty sensations, burning sensations, and redness following prolonged digital screen use [4].

The most crucial step in treating computer vision syndrome is to eliminate the underlying cause of the symptoms. Many of the symptoms of computer vision syndrome can be avoided with proper usage strategies. Poor seating posture, an inappropriate viewing distance, an improper viewing angle, screen brightness, poor lighting, and an imbalance of light between the computer screen and the surroundings are all factors that contribute to computer vision syndrome.

With reports of 4-8% worse performance on work tasks as a result of visual issues, CVS has been dubbed arguably the most significant contemporary occupational hazard [5]. University students have an 89.9% prevalence of CVS, which increases with more than two hours of computer use [6]. Numerous studies revealed that workers in offices and students at colleges had a high prevalence of CVS. One study from 2016 found that 67.4% of computer office workers had this disease, with females having a higher prevalence [7]. In Malaysia, a study by the National Institute of Occupational Safety and Health (NIOSH) found that employees who used computers at work reported lower back pain, neck pain, and shoulder pain in 61.4% of cases and eyestrain in 70.6% of cases [8]. A different study on 416 engineering and medical students in India was conducted in 2014, with prevalence rates of 81.9% and 78.6%, respectively [9]. Additionally, a study conducted on bankers in 2016 revealed a prevalence of 73% as well as the presence of certain risk factors like poor room lighting and an insufficient distance between the eyes and the screen [10]. Thomson reported in a CVS review that as many as 90% of computer users may experience CVS symptoms after prolonged computer use [11].

Despite the fact that computer vision syndrome is readily avoidable [12,13], the condition affects more than 60 million people globally, and one million new cases are diagnosed each year [14,15]. Today, nearly all institutions, including homes, colleges, and universities, frequently utilize computers. Using computers has become a necessity in the 21st century. However, even three hours of use every day increases the chance of developing computer vision syndrome. CVS is not well-known or recognized among university students, and there is a high prevalence of poor posture (shoulder hunching) and neck pain among them. As a result, research is needed to highlight the importance of CVS and the long-term consequences if it is not addressed properly, as well as to educate students about this health issue.

The available research fails to sufficiently address CVS, and there are few research efforts that look at African nations. Even though numerous studies have shown a link between continued computer use, bad posture, and a variety of musculoskeletal pains, the majority of them have only looked at Western populations. The impact of electronic device use on the well-being of African and Sudanese individuals, particularly among university students, has hardly ever been the subject of research.

The primary objective of this study was to estimate the prevalence of symptoms of computer vision syndrome and the factors that contribute to them among medical students at the University of Khartoum. The secondary objective was to examine practices and knowledge related to avoiding computer vision syndrome.

## Materials and methods

This was an observational and descriptive facility-based cross-sectional study conducted among medical students at the University of Khartoum, Sudan, in February 2022. Male and female medical students from all six undergraduate academic years were eligible to participate in this study.

A minimum sample size of 131 was calculated using OpenEpi version 3, using the prevalence of previous studies, a 95% confidence level, an alpha error of 0.05, and a margin of error of 5% [6,16]. Stratified random sampling was used to select 30 students from each of the six academic years using student index numbers obtained from the registrar’s office. One hundred eighty online surveys were sent to the randomly selected students; 149 students completed the questionnaire, yielding a non-response rate of 17.2%.

A validated, structured, closed-ended, self-administered questionnaire (CVS-Q) was used [17]. The questionnaire contained 15 questions that took about 5 minutes to complete. It included questions about sociodemographic characteristics (gender and academic batch number), questions about each recognized CVS symptom (dry eyes, headache, burning eye sensations, blurry vision, eye redness, and neck and shoulder discomfort), and this included each symptom's degree of severity, classified as mild, moderate, or severe. Additionally, it covered factors such as screen-to-eye distance, sitting posture, screen filters, screen brightness, ambient lighting, awareness of the 20-20-20 rule (every 20 minutes take a 20-second break and look at an object 20 feet away), time spent on electronic devices every day, breaks taken while using a device, the frequency and length of those breaks, and the presence of eye disease.

Data were entered into Microsoft Excel spreadsheets and then analyzed using the SPSS Statistics software version 25 (Armonk, NY: IBM Corp.). Simple descriptive statistics (percentages and frequencies) were calculated. The association between the variables was examined and measured using the odds ratios (ORs) and Pearson's chi-square test. A 95% confidence interval (CI) and a p-value of less than 0.05 were utilized for precision and statistical significance.

Ethical approval was granted by the Research Ethics Committee of the Faculty of Medicine, University of Khartoum. Prior to taking part in the study, every participant provided informed consent. Participation was voluntary; no incentives or rewards were offered to the participants. Strict confidentiality of the data was upheld, as no personal data was collected and every questionnaire remained anonymous.

## Results

A total of 180 questionnaires were sent to randomly selected medical students; 149 of the questionnaires were completed, yielding a non-response rate of 17.2%. Female participants made up 58.4% of the sample, while males made up 41.6% of the sample. One hundred forty (94%) students reported experiencing at least one symptom of computer vision syndrome, and 72.4% of them reported experiencing at least three symptoms.

Most reported symptoms were mild. Neck and shoulder pain was the most commonly reported symptom (78.5%), followed by headache (70.5%), while the least reported symptom was eye redness (36.2%). The most common severe symptom was neck and shoulder pain (7.4%), whereas eye redness was the least reported severe symptom (1.3%) (Table 1).

**Table 1:** Prevalence of self-reported computer vision syndrome symptoms among participants (n=149). CVS: computer vision syndrome

CVS symptoms	None, no. (%)	Mild, no. (%)	Moderate, no. (%)	Severe, no. (%)
Headache	44 (29.5%)	60 (40.3%)	41 (27.5%)	4 (2.7%)
Burning eye sensation	45 (30.2%)	62 (41.6%)	34 (22.8%)	8 (5.4%)
Eye redness	95 (63.8%)	37 (24.8%)	15 (10.1%)	2 (1.3%)
Blurred vision	88 (59.1%)	42 (28.2%)	16 (10.7%)	3 (2.0%)
Dry eyes	77 (51.7%)	43 (28.9%)	26 (17.4%)	3 (2.0%)
Neck and shoulder pain	32 (21.5%)	64 (43.0%)	42 (28.2%)	11 (7.4%)

The majority of students (81.2%) used electronic devices for five or more hours a day, and the most common posture while using electronic devices was lying down, as reported by 54.4% of the students. In this study, 68.0% of participants admitted to sitting closer to the screen than the suggested 40 cm, and only 18.1% were aware of the 20-20-20 rule. The seating position has been found to be significantly associated with the number of symptoms experienced (p=0.012); sitting with a bent back was 4.6 times (95% CI: 1.63-13.21, p=0.004) more likely to cause more than three symptoms when compared to sitting upright with a straight back (Table 2).

**Table 2:** Computer vision syndrome associated factors (n=149). OR: odds ratio; CI: confidence interval; Ref: reference variable

Variable		>3 symptoms (n=73)	≤3 symptoms (n=76)	OR	95% CI for OR
Gender	Male	26 (41.9%)	36 (58.1%)	1.0	Ref.
	Female	47 (54.0%)	40 (46.0%)	1.627	0.843-3.140
Hours of usage	>5 h	62 (51.2%)	59 (48.8%)	1.624	0.703-3.754
	≤5 h	11 (39.3%)	17 (60.7%)	1.0	Ref.
Room illumination	Very bright	3 (33.3%)	6 (66.7%)	1.0	Ref.
	Bright	50 (49.5%)	51 (50.5%)	1.961	0.465-8.274
	Dull	9 (37.5%)	15 (62.5%)	1.200	0.239-6.025
	Dark	11 (73.3%)	4 (26.7%)	5.500	0.912-33.184
Seating position	Straight back	8 (28.6%)	20 (71.4%)	1.0	Ref.
	Bent back	26 (65.0%)	14 (35.5%)	4.643	1.631-13.216
	Lying down	39 (48.1%)	42 (51.9%)	2.321	0.917-5.875
Awareness of the 20-20-20 rule	Yes	14 (51.9%)	13 (48.1%)	1.150	0.499-2.648
	No	59 (48.4%)	63 (51.6%)	1.0	Ref.

Female students generally experienced greater symptom prevalence. A total of 83.9% of female students experienced neck and shoulder pain following the use of an electronic device, in contrast with 71.0% of male students, and headaches were experienced by 77.0% of female students compared with 61.3% of male students. Dry eyes and eye redness did not show any statistically significant difference between male and female participants (Table 3). There was no statistically significant difference between male and female participants in terms of knowledge and practices that aid in the prevention of computer vision syndrome (Table 4).

**Table 3:** Distribution and comparison of the prevalence of computer vision syndrome symptoms among male and female participants (n=149).

Symptom		Males, no. (%)	Females, no. (%)	Chi-square value	p-Value
Headache	No symptoms	24 (38.7%)	20 (23.0%)	18.101	<0.001
	Mild	31 (50.0%)	29 (33.3%)		
	Moderate-severe	7 (11.3%)	38 (43.7%)		
Dry eyes	No symptoms	35 (56.5%)	42 (48.3%)	1.804	0.406
	Mild	18 (29.0%)	25 (28.7%)		
	Moderate-severe	9 (14.5%)	20 (23.0%)		
Burning eye sensation	No symptoms	23 (37.1%)	22 (25.3%)	7.830	0.020
	Mild	29 (46.8%)	33 (37.9%)		
	Moderate-severe	10 (16.1%)	32 (36.8%)		
Eye redness	No symptoms	39 (62.9%)	56 (64.4%)	0.054	0.973
	Mild	16 (25.8%)	21 (24.1%)		
	Moderate-severe	7 (11.3%)	10 (11.5%)		
Blurred vision	No symptoms	40 (64.5%)	48 (55.2%)	8.715	0.013
	Mild	20 (32.3%)	22 (25.3%)		
	Moderate-severe	2 (3.2%)	17 (19.5%)		
Neck and shoulder pain	No symptoms	18 (29.0%)	14 (16.1%)	23.806	<0.001
	Mild	36 (58.1%)	28 (32.2%)		
	Moderate-severe	8 (12.9%)	45 (51.7%)		

**Table 4:** Comparison of male and female participants' knowledge and practices regarding computer vision syndrome (n=149).

Practice		Males, no. (%)	Females, no. (%)	Chi-square value	p-Value
Hours of usage	>5 h	51 (82.3%)	70 (80.5%)	0.077	0.782
	≤5 h	11 (17.7%)	17 (19.5%)		
Distance (in cm)	<40	39 (62.9%)	62 (71.3%)	1.622	0.654
	40-76	14 (22.6%)	16 (18.4%)		
	>76	2 (3.2%)	1 (1.1%)		
	Does not know	7 (11.3%)	8 (9.2%)		
Screen brightness	Very bright	3 (4.8%)	1 (1.1%)	4.967	0.174
	Bright	21 (33.9%)	30 (34.5%)		
	Dull	33 (53.2%)	40 (46.0%)		
	Very dull	5 (8.1%)	16 (18.4%)		
Room illumination	Very bright	3 (4.8%)	6 (6.9%)	3.910	0.271
	Bright	43 (69.4%)	58 (66.7%)		
	Dull	7 (11.3%)	17 (19.5%)		
	Dark	9 (14.5%)	6 (6.9%)		

## Discussion

This study investigated the prevalence of self-reported symptoms of computer vision syndrome and its associated factors among medical students at the University of Khartoum in February 2022. Females made up 58.4% of the sample, while males made up 41.6%; this is likely due to the fact that females make up the majority of medical students at the University of Khartoum. A total of 140 (94%) students reported experiencing at least one symptom of computer vision syndrome; this prevalence is similar to that reported by Gammoh in Jordan, which found CVS prevalence among university students to be 94.5% [18].

The prevalence reported in this study was higher than the prevalence reported in most other similar studies, which was 62.3% among graduate students in Peru [19], 73.0% among bank workers in Ethiopia [10], 74.3% among medical students in China [20], 77.0% among school students in China during coronavirus disease 2019 (COVID-19) [21], 77.5% among medical students in India [22], 78.6% among medical students in India [9], 86.0% among medical students in Egypt [23], 89.9% among students in Malaysia [6], 97.3% among health sciences students in Saudi Arabia [16], and 97.9% among students in Thailand [24].

In this study, 108 (72.4%) students reported experiencing at least three symptoms of CVS, which is higher than the 52.7% reported in Saudi Arabia [17]. The most commonly experienced symptom was neck and shoulder pain (78.5%), followed by headache (70.5%), while the least common symptom was eye redness (36.2%). This is similar to the study in Saudi Arabia, which also found the most commonly experienced symptom was neck and shoulder pain with a prevalence of 82.2% [17]. Similarly, a study in Jamaica found neck pain to be the most commonly experienced symptom (75.1%) [25]. The second most common symptom reported in this study was headache, with a prevalence of 70.5% In Saudi Arabia and Malaysia, headaches were the most frequently reported symptom, with a prevalence of 68.0% and 19.7%, respectively [6,16]. While other studies have reported the prevalence of headaches ranging from 23.0% in Ethiopia [10], 26.0% in Egypt [23], 61.0% in Japan [15], 66.5% in Saudi Arabia [17], and 82.1% in India [26], these reports suggest that headaches are a common symptom due to frequent computer use.

This study also documents the widespread prevalence of other ocular symptoms, such as dry eyes and a burning sensation in the eyes. Burning eye sensations were one of the most commonly reported severe symptoms in this study, with a total prevalence of 69.8% and 5.4% reporting it as severe. Other studies have reported a slightly lower prevalence of burning eye sensations, which ranged from 58.3% in Saudi Arabia [17] to 61.9% in Jamaica [25] and 62% in Saudi Arabia [16].

This study found the prevalence of dry eyes to be 48.3%, which is the same as a study in Saudi Arabia, which reported a prevalence of 48.3% [16]. This prevalence is higher than the one reported in Egypt (28%), where dryness was the most common symptom [23], but lower than the one reported among medical students in China (72.97%) [20]. A study in Indonesia among high school students found that 19.1% of participants had dry eyes with moderate exposure to mobile devices, and 56.4% of the students had dry eyes with heavy exposure. Results show that even low levels of exposure to mobile devices can increase the risk of developing evaporative dry eyes, one of the symptoms of CVS, in children with normal tear production [27].

Blurry vision has been linked to computer use in several studies [6,23,25]. This symptom was reported as mild, moderate, or severe by 40.9% of study participants. The least common symptom in this study was eye redness, which had a prevalence of 36.2%; this is lower than the prevalence reported by similar studies, which was 51.0% in Saudi Arabia [16] and (61.2%) in India [26]. Most reported symptoms were mild. The most common severe symptom was neck and shoulder pain (7.4%), while eye redness was the symptom that was least frequently reported as being severe (1.3%).

To assess the factors that contribute to CVS, two following categories were formed: students who had more than three symptoms and students who had three symptoms or fewer. The seating position was the only factor that was found to be significantly associated with the number of symptoms experienced in this study (p=0.012); sitting with a bent back was 4.6 times more likely to cause more than three symptoms when compared to sitting upright with a straight back (OR=4.643; 95% CI: 1.63-13.21, p=0.004). The majority of students (54.4%) reported lying down most of the time they are using electronic devices, and only 18.8% used the correct position of sitting upright with a straight back. A study in Jamaica found that severe eye strain occurred in 63.0% of those who looked down at the device compared with 21.0% of those who kept the device at eye level, which suggests that correct posture and seating position play an important role in preventing CVS occurrence [25].

This study found that the majority of students (81.2%) used electronic devices for five or more hours a day. Reddy et al.'s study in Indonesia found a significant correlation between the occurrence of CVS symptoms and daily computer use of more than two hours [6]. In this study, 68.0% of the medical students admitted to sitting closer to the screen than the suggested 40 cm, which is strongly associated with CVS. Numerous studies have linked closer proximity to screens with a higher incidence of CVS symptoms [28,29].

The majority of students in this study (81.9%) were unaware of the 20-20-20 rule (every 20 minutes look at an object 20 feet away for 20 seconds), which is similar to what was reported in a study in Saudi Arabia, which found that 77.3% of students were unaware of the 20-20-20 rule [17]. Numerous studies have found that occasionally looking at distant objects while using a digital device is strongly linked to suffering fewer symptoms of CVS [6,17]. However, this study did not find a statistically significant difference in the number of symptoms experienced between participants who knew the 20-20-20 rule and those who didn't, suggesting that knowledge may not always translate into practice. A total of 49% of the students used devices with dull screens, and 68% used devices in bright rooms, which are both good practices. In terms of knowledge and practices that help in the prevention of computer vision syndrome, there was no statistically significant difference between male and female participants.

Regarding the study's limitations, self-reporting is an important challenge because participants may either underestimate or overestimate their symptoms. The ability to generalize is also limited to university students, as the study's participants don't accurately represent the general public. The study nonetheless provides a significant contribution despite its drawbacks due to the scarcity of comparable data in the region.

## Conclusions

This study found a very high prevalence of computer vision syndrome symptoms among medical students at the University of Khartoum. Neck and shoulder pain, followed by headaches, were the most frequently reported symptoms, while eye redness was the least prevalent. Females generally reported higher symptom prevalence than males. The seating position has been found to be significantly associated with the number of CVS symptoms experienced. Most students had bad habits regarding seating position, hours of usage of electronic devices, and screen-to-eye distance. And most were unaware of the 20-20-20 rule. These findings suggest that students are not aware of the general precautions for safe computer and other digital device use.

Universities are strongly encouraged to organize awareness campaigns about computer vision syndrome and its contributing factors, with a particular emphasis on the proper use of electronic devices for students and the general public. These campaigns could take the form of lectures and educational posters that encourage good practices and enable the safe use of computers and other electronic devices.
